# The Effect of Work-Related Use of Information and Communication Technologies on Employees’ Work Goal Progress and Fatigue: Based on the Transactional Model of Stress

**DOI:** 10.3390/bs15091197

**Published:** 2025-09-03

**Authors:** Xiangping Zhan, Pengfei Zhang, Hongyu Ma

**Affiliations:** 1 School of Psychology, Central China Normal University, Wuhan 430079, China; zhanxp@mails.ccnu.edu.cn (X.Z.); zpfpsy_ccnu@163.com (P.Z.); 2 Key Laboratory of Adolescent Cyberpsychology and Behavior (CCNU), Ministry of Education, Wuhan 430079, China

**Keywords:** work-related use of information and communication technologies after hours, cognitive appraisal, work goal progress, fatigue, family supportive supervisor behaviors

## Abstract

The rapid evolution of information and communication technologies (ICTs) has made after-hours work-related ICTs (W_ICTs) use commonplace. The double-edged sword effects of W_ICTs have been widely concerned by researchers, but the role of cognitive appraisal has not been fully investigated. Based on the Transactional Model of Stress, this study explores the underlying mechanism and boundary condition of W_ICTs on work goal progress and fatigue. The hypotheses were tested using 200 two-wave employees’ data. The results showed the following: W_ICTs could improve work goal progress through challenge appraisal; Family Supportive Supervisor Behaviors (FSSB) could strengthen the positive relationship between W_ICTs and challenge appraisal while indirectly strengthening the positive relationship between W_ICTs and work goal progress and the negative relationship between W_ICTs and fatigue. This study revealed the positive impact of W_ICTs and the strengthening effect of FSSB on the W_ICTs–challenge appraisal relationship.

## 1. Introduction

With the rapid advancement of modern information and communication technologies, the boundary between work and family is becoming increasingly blurred. The widespread use of intelligent electronic devices has made it common for employees to handle work tasks outside of regular working hours, and work-related use of information and communication technologies (W_ICTs) after hours has become a prevalent phenomenon. W_ICTs have attracted widespread attention from the researchers and practitioners of the organizational behavior field, which brings work into non-work domains, affecting employees’ work outcomes and non-work recovery. Work goal progress is a judgment made by employees after comparing the current level with the expected level of goal achievement (Nie et al., 2021), which directly reflects the work outcomes brought about by W_ICTs. Fatigue is the experience of depletion of self-control resources (Hagger et al., 2010), reflecting a low-level recovery of individuals. A meta-analysis by Thörel et al. (2022) showed that W_ICTs have negative impacts on employees’ recovery; however, when it comes to work, W_ICTs may be perceived as a resource, thereby promoting work engagement. Based on this, the present study takes work goal progress and fatigue as indicators to reflect the positive work outcomes and negative non-work outcomes influenced by W_ICTs.

W_ICTs own the “Empowerment-Enslavement Paradox” (Jarvenpaa & Lang, 2005), which is specifically reflected in three aspects: autonomy, social connectedness, and productivity (Day et al., 2019). On the one hand, W_ICTs enhance employees’ perceived autonomy, flexibility, and connectivity in terms of work schedule and location. This helps to meet their basic psychological needs and, in turn, improves work performance (Ďuranová & Ohly, 2016; Xie et al., 2018; Day et al., 2019). On the other hand, W_ICTs place employees in a situation where they are constantly required to respond to job demands. This leads to work–family conflict, a lack of detachment, and health impairment (Park et al., 2020; Thörel et al., 2022). Researchers suggested that examining the impact of W_ICTs on employee well-being from a solely positive or negative perspective is a key reason for inconsistent conclusions regarding W_ICTs’ effects (Ma et al., 2016; Wang et al., 2019). Thus, it is essential to adopt a heterogeneous perspective to clarify the impact of W_ICTs.

Based on a heterogeneity perspective and employing different theories, researchers have explored the double-edged sword effect of W_ICTs. The Work–Family Border Theory shows that W_ICTs can cause both work–family conflict and enrichment, affecting well-being via social interactions (Ma et al., 2016). The Conservation of Resources Theory indicates that W_ICTs can create resource gain or loss spirals, leading to different outcomes (Richardson & Thompson, 2012; Ward & Steptoe-Warren, 2013). The Job Demands-Resources Model views W_ICTs as either a demand or resource, with Ter Hoeven et al. (2016) finding it increases well-being through a motivational pathway (availability and efficiency) but decreases it through a depleted pathway (interruption and unpredictability). However, Day et al. (2019) proposed that the category of W_ICTs as a resource or demand varies among individuals. Wang et al. (2019) proposed that evaluating the relationship between W_ICTs and personal goals is important to clarify their impact on well-being.

The Transactional Model of Stress (TMS; Lazarus & Folkman, 1984) provides a favorable framework for us to understand the double-edged sword effect of W_ICTs. This theory holds that the appraisal of a stimulus as a benefit or threat determines individuals’ reaction to it. When individuals appraise the stressor as a challenge, which has the potential to promote growth, development, and well-being, it is more likely to bring positive outcomes; however, if they interpret the stressor as a hindrance, which causes loss, constraint, or harm, it is more likely to lead to negative outcomes (LePine et al., 2016). As a neutral stimulus, W_ICTs trigger specific behavioral responses through individuals’ different cognitive appraisals (Reinke & Ohly, 2021; Braukmann et al., 2018). Research showed that people appraise communication pressure as both a challenge and a hindrance, which have positive and negative effects on work performance, respectively (Lei, 2017). When W_ICTs are related to goal progress, they are more likely to be appraised as challenges, thereby eliciting positive emotions; however, when W_ICTs are related to overload, they are more likely to be appraised as hindrances, leading to negative emotions and low psychological detachment (Reinke & Ohly, 2021). While Reinke and Ohly (2021) applied the TMS to show how specific ICT-use experiences—e.g., perceived goal progress—shape subsequent ICT appraisals, we treat W_ICTs themselves as the primary stimulus within TMS, capable of eliciting either challenge or hindrance appraisals regardless of immediate usage outcomes.

Moreover, the TMS highlights that the cognitive appraisal of a stressor is influenced not only by the stimulus itself but also by the available resources. These resources can be at both the individual and organizational levels and shape individuals’ focus on the characteristics (i.e., positive or negative aspects) of the stressor (F. Liu et al., 2022). Research on W_ICTs has yet to examine how leadership shapes employees’ appraisal, although mounting evidence shows that leader behaviors can reshape employees’ interpretations of challenge stressors (LePine et al., 2016; F. Liu et al., 2022). This study proposes Family Supportive Supervisor Behaviors (FSSBs) as a crucial organizational resource that may shape employees’ appraisal of W_ICTs. FSSB refers to a supervisor’s supportive behaviors towards employees’ family (or non-work) needs while balancing organizational goals (Hammer et al., 2009). Compared with other supportive leadership behaviors, FSSB is more targeted in helping employees maintain work–family balance (Kossek et al., 2011) and may be a more explanatory variable at the organizational level, influencing employees’ assessment of W_ICTs. Employees who perceive a high level of FSSB may be more likely to appraise W_ICTs as a challenge rather than a hindrance; this is because they may receive supervisor support to manage the potential negative effects of W_ICTs, thereby enhancing their positive impacts.

In sum, this study examines the dual effects of W_ICTs and identifies key factors that enhance positive and buffer negative impacts, providing a comprehensive understanding of W_ICTs’ influence on employees’ work and non-work outcomes. The main contributions are the following: (1) Explores how W_ICTs differently affect work and non-work outcomes using TMS, addressing prior research on cognitive appraisal as a mechanism. (2) Examines both work and non-work outcomes, clarifying W_ICTs’ impacts. (3) Investigates the moderating role of FSSB, offering suggestions for managing W_ICTs practices.

### 1.1. The Role of Cognitive Appraisal in the Double-Edged Sword Effect of W_ICTs

#### 1.1.1. The Mediating Role of Challenge Appraisal

W_ICTs can elicit challenge appraisals. Firstly, W_ICTs enhance job autonomy and flexibility, increasing employees’ sense of control over their work and providing convenience for continuing work after hours. Engaging in additional work in the evening can alleviate time pressure in the following workday (Ďuranová & Ohly, 2016). Secondly, W_ICTs have the potential to promote employee growth and development by facilitating progress toward work goals, thereby improving work performance (Nie et al., 2021). When employees appraise stressors as a challenge, it can motivate them to work harder (C. Liu & Li, 2018) and enhance work performance (LePine et al., 2016). Research has found that the challenge appraisal of communication pressure can positively predict work performance (Lei, 2017). Moreover, challenge appraisal can reduce fatigue by increasing intrinsic motivation (Parker et al., 2019). Autonomous motivation for W_ICTs can also predict higher levels of recovery and psychological detachment (Ohly & Latour, 2014). When individuals appraise W_ICTs as challenges, they can quickly detach from work after engaging in W_ICTs, thereby reducing fatigue. Therefore, this study proposes Hypothesis 1-1: W_ICTs have a positive effect on work goal progress via challenge appraisal (1-1a) and a negative effect on fatigue via challenge appraisal (1-1b).

#### 1.1.2. The Mediating Role of Hindrance Appraisal

W_ICTs may lead to hindrance appraisals. Firstly, W_ICTs reflect an increase in work tasks and extended working hours, while placing employees under the stress of being “on call” at all times, which depletes their physical and mental resources (He & Yu, 2020). Secondly, W_ICTs occupy employees’ leisure time, which is not conducive to their psychological detachment and the replenishment of energy consumed during daytime work (Ward & Steptoe-Warren, 2013). Previous studies have shown that hindrance appraisal of a job demand led to insufficient work motivation and reduced work performance (Lei, 2017; C. Liu & Li, 2018). If employees perceive W_ICTs as burden rather than linking them to the achievement of work goals, they are less likely to utilize W_ICTs to advance their work goals. Moreover, when employees appraise W_ICTs as hindrances, their recovery experience is also affected. Reinke and Ohly (2021) found that employees’ negative appraisal of W_ICTs reduces psychological detachment, which in turn leads to fatigue (Lee et al., 2021). Therefore, this study proposes Hypothesis 1-2: W_ICTs have a negative effect on work goal progress via hindrance appraisal (1-2a) and a positive effect on fatigue via hindrance appraisal (1-2b).

### 1.2. The Moderating Role of FSSB

Recognizing and responding to employees’ family-related needs—such as caregiving responsibilities, parental leave, and access to childcare—is a foundational component of building a sustainable workplace (Samul, 2020). FSSB refers to the behaviors exhibited by supervisors that provide support for employees’ family-related needs (Hammer et al., 2009), which contains four dimensions: emotional support, instrumental support, role modeling, and innovative management. As an organizational resource, FSSB includes both emotional and instrumental support that supervisors offer to employees in the family domain, which provides employees with supportive resources to fulfill their family responsibilities (Song et al., 2019). Park et al. (2020) demonstrated in a sample of elementary school teachers that principals’ work–family support, by increasing teachers’ boundary control, buffers the impact of ICT demands on negative work rumination. Leaders have a certain ability to allocate organizational resources; therefore, the resources provided by leaders are important factors influencing the assessment of employees’ coping potential (Jiang & Wang, 2022). When FSSB is high, leaders actively support employees’ family needs, and even delayed responses are tolerated. In this context, W_ICTs are experienced as autonomous activities that generate little pressure, prompting employees to appraise them as challenges. Conversely, when FSSB is low, leaders press employees for rapid replies, turning W_ICTs into controlled demands. The heightened pressure leads employees to appraise W_ICTs as hindrances. Therefore, this study proposes Hypothesis 2-1: FSSB can strengthen the relationship between W_ICTs and challenge appraisal (2-1a) and buffer the relationship between W_ICTs and hindrance appraisal (2-1b).

Furthermore, by integrating Hypotheses 1 and 2, this study proposes the moderated mediating model with FSSB as the moderator. Hypothesis 3-1: FSSB can strengthen the indirect relationship between W_ICTs and work goal progress via challenge appraisal (3-1a) and the indirect relationship between W_ICTs and fatigue via challenge appraisal (3-1b); it can also buffer the indirect relationship between W_ICTs and work goal progress via hindrance appraisal (3-1c) and the indirect relationship between W_ICTs and fatigue via hindrance appraisal (3-1d).

**Figure 1:**
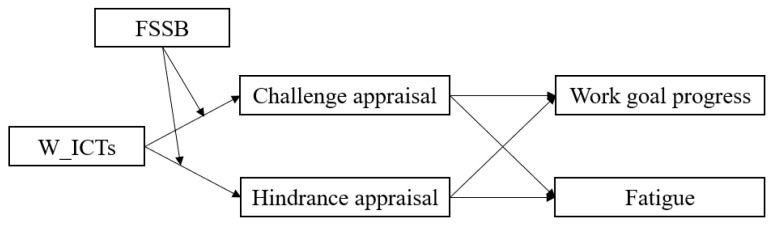
The hypothesized model.

The hypothesized model of this study is shown in Figure 1.

## 2. Materials and Methods

### 2.1. Participants

Participants—full-time employees from various Chinese organizations—were recruited via snowball sampling. Data were collected online through the Wenjuanxing platform at two time points with a six-week interval. At Time 1, employees completed questionnaires on W_ICTs, challenge and hindrance appraisal of W_ICTs, FSSB, and demographic information, with 486 valid questionnaires collected. At Time 2, data on work goal progress and fatigue were collected, yielding 230 valid questionnaires. Ultimately, 200 valid matched questionnaires were obtained, with an effective response rate of 41.15%. The sample consisted of 63 men (31.5%) and 137 women (68.5%); 156 participants (78%) were between 18 and 30 years old, 14 (7%) were between 31 and 40 years old, 17 (8.5%) were between 41 and 50 years old, and 13 (6.5%) were over 51 years old. Regarding marital status, 71 were single (35.5%), 79 were in a relationship (39.5%), 48 were married (24%), and 2 were divorced (1%). Only 18 participants (9%) had children under 18 years old.

### 2.2. Measures

All scales were translated into Chinese using Brislin’s (1980) translation and back-translation procedure, based on well-established scales. All scales were rated on a 5-point Likert scale, ranging from 1 (strongly disagree) to 5 (strongly agree).

#### 2.2.1. W_ICTs

W_ICTs were assessed with a 3-item scale by Ma et al. (2016). An example item is “How often do you use ICTs to connect with related personnel for work purpose after hours”. The Cronbach’s α coefficient for this scale was 0.88.

#### 2.2.2. Cognitive Appraisal

The measurement of cognitive appraisal was adapted from LePine et al. (2016) and included 3 items for each kind of appraisal. Example items are “Overall, using work-related information and communication technologies after hours promotes my personal achievement (challenge appraisal)” and “Overall, using work-related information and communication technologies after hours hinders my personal achievement (hindrance appraisal)”. The Cronbach’s α coefficients for the challenge and hindrance appraisal subscales were 0.85 and 0.88, respectively.

#### 2.2.3. FSSB

FSSB was assessed with a 4-item short-form scale by Hammer et al. (2013). An example item is “Your supervisor makes you feel comfortable talking to him/her about your conflicts between work and non-work”. The Cronbach’s α coefficient for this scale was 0.84.

#### 2.2.4. Work Goal Progress

The measurement of work goal progress was adapted from Koopman et al. (2016) and included 3 items. An example item is “Using work-related information and communication technologies after hours has brought me closer to my work goals”. The Cronbach’s α coefficient for this scale was 0.91.

#### 2.2.5. Fatigue

Fatigue was assessed with a 5-item scale by McNair et al. (1971). An example item is “Recently, I felt fatigued”. The Cronbach’s α coefficient for this scale was 0.97.

#### 2.2.6. Control Variables

According to prior research (Xie et al., 2018; Eichberger et al., 2022), gender and workload may influence the recovery level under the W_ICTs context and were included as control variables. Workload was assessed with a 5-item perceived work demand scale by Boyar et al. (2007). An example item is “I am given a lot of work to do”. The Cronbach’s α coefficient for this scale was 0.88.

### 2.3. Data Analysis

First, confirmatory factor analysis (CFA) was conducted to examine the discriminant validity of study variables by Mplus 8.3. Next, descriptive statistics were calculated using SPSS 23.0. Finally, the moderated mediation analysis was performed using Model 7 in PROCESS v4.0 of SPSS 23.0.

## 3. Results

### 3.1. Common Method Bias Test

The results of CFA indicated that the hypothesized six-factor model fit the data best (χ^2^ = 304.70, *df* = 174, RMSEA = 0.05, SRMR = 0.04, CFI = 0.97, TLI = 0.96), which was superior to other alternative models, suggesting good discriminant validity. Additionally, the Harman single-factor test was employed to assess common method bias; it was found that the largest factor accounted for 29.1% of the total variance under the unrotated condition, which is below the 40% criterion, indicating that there is no serious common method bias in this study.

### 3.2. Descriptive Statistics and Correlation Analysis

The means, standard deviations, and correlations among variables are presented in Table 1. W_ICTs were not significantly correlated with challenge appraisal (*r* = 0.12, *p* = 0.10) or hindrance appraisal (*r* = 0.09, *p* = 0.21). Challenge appraisal was positively correlated with work goal progress (*r* = 0.40, *p* < 0.01) and negatively correlated with fatigue (*r* = −0.24, *p* < 0.01). Hindrance appraisal was positively correlated with fatigue (*r* = 0.24, *p* < 0.01) and negatively correlated with work goal progress (*r* = −0.23, *p* < 0.01).

### 3.3. The Mediating Role of Cognitive Appraisal

The full model path analysis results are presented in Table 2. The regression analysis revealed that, after controlling for gender and workload, W_ICTs significantly and positively predicted challenge appraisal (*b* = 0.21, *p* < 0.01). Challenge appraisal, in turn, had a significant positive effect on work goal progress (*b* = 0.30, *p* < 0.001) and a significant negative effect on fatigue (*b* = −0.16, *p* < 0.05). The mediating effect analysis showed that the indirect effect of W_ICTs on work goal progress through challenge appraisal was 0.06, with a 95% Confidence Interval (CI) of [0.01, 0.13], which did not include zero. Conversely, the indirect effect of W_ICTs on fatigue through challenge appraisal was −0.03, with a 95% CI of [−0.09, 0.001], which included zero. Therefore, Hypothesis 1-1a was supported, while Hypothesis 1-1b was not supported.

Additionally, W_ICTs did not significantly affect hindrance appraisal (*b* = 0.01, *p* = 0.93), and hindrance appraisal did not significantly influence work goal progress (*b* = −0.08, *p* = 0.16), but it did have a significant positive effect on fatigue (*b* = 0.15, *p* < 0.05). Thus, neither Hypothesis 1-2a nor Hypothesis 1-2b was supported.

### 3.4. The Moderating Role of FSSB

**Figure 2:**
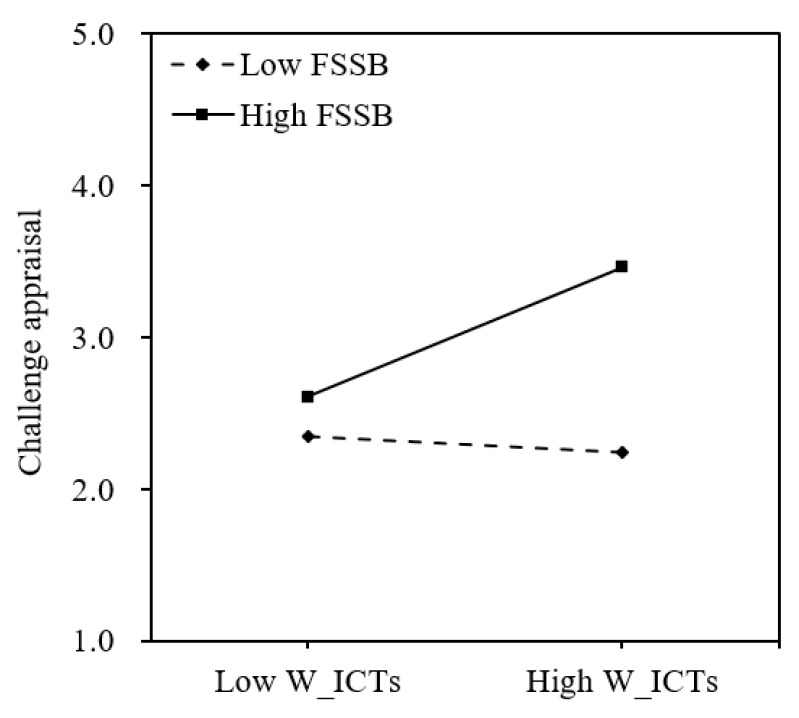
The moderating effect of FSSB on the relationship between W_ICTs and challenge appraisal.

As indicated in Table 2, the interaction between W_ICTs and FSSB significantly and positively influenced challenge appraisal (*b* = 0.32, *p* < 0.001), yet it did not have a significant impact on hindrance appraisal (*b* = −0.12, *p* = 0.22). Further simple slope analysis (Figure 2) revealed that among employees with high levels of FSSB (+1 *SD*), W_ICTs had a significant positive effect on challenge appraisal (*b* = 0.49, *t* = 4.46, 95% CI = [0.27, 0.70]). In contrast, for employees with low levels of FSSB (−1 *SD*), the effect of W_ICTs on challenge appraisal was insignificant (*b* = −0.06, *t* = −0.56, 95% CI = [−0.28, 0.16]). Therefore, Hypothesis 2-1a was supported, whereas Hypothesis 2-1b was not supported.

### 3.5. The Moderated Mediation Effect Test

Given that FSSB did not moderate the relationship between W_ICTs and hindrance appraisal, Hypotheses 2-2c and 2-2d were not supported. Therefore, only Hypotheses 2-2a and 2-2b were tested. Table 3 shows that the indirect effect of W_ICTs on work goal progress through challenge appraisal was significantly different between the high and low FSSB groups, with a 95% CI of [0.07, 0.29], which did not include zero. Similarly, the indirect effect of W_ICTs on fatigue through challenge appraisal also differed significantly between the high and low FSSB groups, with a 95% CI of [−0.18, −0.002], which did not include zero. Thus, Hypotheses 2-2a and 2-2b were supported.

## 4. Discussion

Based on TMS, this study investigated the impact of W_ICTs on employees’ work (work goal progress) and non-work (fatigue) outcomes through challenge and hindrance appraisals, as well as the moderating role of FSSB in the above processes. Based on 200 two-wave data, the results showed that in terms of cognitive appraisal, employees are more likely to regard W_ICTs as a challenge. W_ICTs can enhance work goal progress by stimulating employees’ challenge appraisal, and this relationship is stronger among employees with high FSSB.

### 4.1. Theoretical Implications

This study advances organizational psychology and digital work behavior research through two core theoretical contributions:

First, this study links W_ICTs with work goal progress and fatigue from a heterogeneity perspective (i.e., double-edged sword effect) and attempts to comprehensively reveal the impact brought by W_ICTs from both work and non-work domains. The results showed that W_ICTs can elicit challenge appraisal, which in turn positively predicts work goal progress. This is consistent with previous research showing that W_ICTs positively predict work outcomes (Nie et al., 2021; Lei, 2017). Shi et al. (2018) demonstrated that W_ICTs can increase employees’ attention to work opportunities, especially for those who are work-oriented. When employees recognize the potential of W_ICTs for personal growth and future development, they are willing to sacrifice leisure time for greater resource gains in the future, leading to more challenge appraisals of W_ICTs. The study found that challenge appraisal of W_ICTs can negatively predict fatigue, although the mediating effect of challenge appraisal was not significant. This may be due to a suppression effect, as the negative indirect effect of W_ICTs on fatigue through challenge appraisal was significant under high FSSB conditions. This finding provides some evidence that W_ICTs can have a positive impact on recovery through challenge appraisal, which helps to reconcile the inconsistent results in previous studies on the impact of W_ICTs on recovery (Ohly & Latour, 2014; Braukmann et al., 2018). Additionally, since the study did not find the direct effect of W_ICTs on hindrance appraisal, it did not reveal the negative path of W_ICTs through hindrance appraisal. It is probably because in the context of Chinese culture, society encourages employees to do extra work for their families, and employees may regard working during non-working hours as self-sacrifice for their families (Galovan et al., 2010). Through W_ICTs, employees can handle their work better and will not make too many negative appraisals of it. In sum, this study adopts the cognitive appraisal perspective to comprehensively understand the impact of W_ICTs on both work and non-work outcomes. It makes an important supplement to the research of W_ICTs from the perspective of the TMS and enriches the application scenarios of the TMS.

Second, this study further reveals the boundary condition that affects employees’ cognitive appraisal. The results showed that FSSB can strengthen the positive relationship between W_ICTs and challenge appraisal. This is aligned with LePine et al. (2016) and F. Liu et al. (2022); effective leadership has been shown to actively reframe employees’ appraisals of stressors. FSSB is a crucial connection chain between organizational support and employee output. As an effective leadership for employees dealing with work–family conflict, FSSB stimulates the “empowerment” aspect of W_ICTs. It equips employees to overcome W_ICT-induced challenges, recasting these demands as catalysts for personal growth and sustaining a positive work state (Shi et al., 2022). This, in turn, promotes work goal progress and has a positive impact on recovery. However, this study did not find the moderating effect of FSSB between W_ICTs and hindrance appraisal. The reason might be that employees viewed W_ICTs as challenges, and external support factors (e.g., FSSB) played a facilitating role by helping employees better cope with the challenge of W_ICTs. However, when employees regarded W_ICTs as obstructions, perhaps individuals’ appraisal would no longer be influenced by external support factors. In sum, considering the issue of work–family boundary management, this study reveals an important organizational boundary condition (FSSB) that facilitates employees to make more challenge appraisals of W_ICTs. By embedding leadership into W_ICTs research, this study not only deepens the theoretical understanding of W_ICTs but also translates this insight into practical approaches for organizations.

### 4.2. Practical Implications

By revealing the relationship between W_ICTs and work goal progress as well as fatigue, this study offers references for organizations on how to manage W_ICTs. First, the study found that cognitive appraisal can serve as a mediating mechanism linking W_ICTs to work and non-work outcomes. This suggests that organizations should help employees view W_ICTs more objectively. Although W_ICTs may affect employees’ recovery process, their potential benefits in advancing work can offset the tension employees feel regarding W_ICTs. Supervisors can openly discuss with employees the positive and negative impacts of W_ICTs, guide employees to see the beneficial side of W_ICTs for personal development, or take measures to motivate employees who proactively engage in W_ICTs. This can encourage employees to actively utilize the organizational supportive resources and better achieve organizational goals. Second, the study found that FSSB plays a significant role in helping employees form challenge appraisal of W_ICTs, which in turn leads to positive work and non-work outcomes. This suggests that organizations should emphasize training managers in FSSB and improving its effectiveness. For example, when subordinates experience work–family conflict due to W_ICTs, a supervisor can better provide emotional comfort and support, offer more scientific and effective methods for handling work–family conflict, and set a good example for them. When employees master the methods to deal with W_ICTs, they will realize the potential of W_ICTs for personal growth and work achievement, and thus form more challenge appraisals.

### 4.3. Limitations and Future Research Directions

The study has the following limitations. First, all the measurements in this study were self-reported, which may be subject to common method bias. Future research should attenuate common method bias by triangulating multiple sources, such as obtaining employees’ objective work performance indicators from organizations or using physiological indicators to reflect employees’ recovery status. Second, the study’s final sample comprised only 200 participants, limiting the external validity of the findings. Future research should recruit a larger and more diverse sample to strengthen generalizability. Third, this study only found a predictive effect of W_ICTs on challenge appraisal. According to TMS, the same stressor can be appraised as both a challenge and a hindrance, and a previous study on the appraisal of W_ICTs also found this result (Reinke & Ohly, 2021). Future research should refine the measurement of W_ICTs to provide evidence for the hindrance appraisal of W_ICTs. Finally, some variables in this study may have other forms of influencing relationships. For example, research has found that work goal progress of W_ICTs can promote positive appraisal of W_ICTs (Reinke & Ohly, 2021), and psychological detachment can predict subsequent ICT use and task progress (Heissler et al., 2022). Future research can explore other possible influencing relationships between variables, such as using longitudinal data to investigate the bidirectional relationship between variables.

## 5. Conclusions

This study investigates the mechanism by which W_ICTs affect work goal progress and recovery from the perspective of cognitive appraisal. Using data from 200 employees at two time points, a dual-path model was constructed to examine how W_ICTs influence work goal progress and recovery. The moderating role of FSSB and the moderated mediating model were also tested. Results indicated that W_ICTs positively predict challenge appraisal but are unrelated to hindrance appraisal. FSSB strengthens the positive relationship between W_ICTs and challenge appraisal. Specifically, the higher the level of employees’ FSSB, the more W_ICTs can lead to challenge appraisal. Moreover, when FSSB is high, W_ICTs more effectively promote challenge appraisal, which in turn enhances work goal progress.

## Figures and Tables

**Table 1:** Means, standard deviations, and correlation coefficients of study variables.

Variables	*M*	*SD*	1	2	3	4	5	6	7
1. Gender	0.69	0.47							
2. Workload	3.86	0.67	0.03						
3. W_ICTs	3.27	0.88	0.04	0.41 **					
4. FSSB	3.14	0.86	0.03	−0.04	−0.08				
5. Challenge appraisal	2.64	1.04	0.00	−0.03	0.12	0.40 **			
6. Hindrance appraisal	3.08	1.07	0.05	0.16 *	0.09	−0.15 *	−0.36 **		
7. Work goal progress	3.11	0.87	−0.03	−0.01	0.05	0.22 **	0.40 **	−0.23 **	
8. Fatigue	2.81	0.94	0.15 *	0.08	−0.02	−0.27 **	−0.24 **	0.24 **	−0.12

Note: *N* = 200. Gender: 0 = male, 1 = female. W_ICTs = work-related use of information and communication technologies, FSSB = family supportive supervisor behaviors. * *p* < 0.05, ** *p* < 0.01.

**Table 2:** The results of the full model path analysis.

Variables	Challenge Appraisal		Hindrance Appraisal		Work Goal Progress		Fatigue	
	*b*	*SE*	*b*	*SE*	*b*	*SE*	*b*	*SE*
*Control variables*								
Gender	−0.00	0.14	0.02	0.15	−0.04	0.12	0.29 *	0.14
Workload	−0.16	0.11	0.30 **	0.12	0.01	0.10	0.09	0.11
* Independent variable *								
W_ICTs	0.21 **	0.08	0.01	0.09	0.01	0.07	−0.05	0.08
* Mediating variables *								
Challenge appraisal					0.30 ***	0.06	−0.16 *	0.07
Hindrance appraisal					−0.08	0.06	0.15 *	0.07
* Moderating variable *								
FSSB	0.43 ***	0.30	−0.09	0.09				
* Interaction term *								
W_ICTs × FSSB	0.32 ***	0.09	−0.12	0.09				
*R* ^2^	0.26 ***		0.18 ***		0.17 ***		0.11 ***	

Note: *N* = 200. W_ICTs = work-related use of information and communication technologies, FSSB = family supportive supervisor behaviors. * *p* < 0.05, ** *p* < 0.01, *** *p* < 0.001.

**Table 3:** The results of the test for the moderated mediating effect.

Path	Groups	*Estimates*	* SE *	95% CI
W_ICTs → Challenge appraisal → Work goal progress	High FSSB	0.15	0.05	[0.06, 0.25]
	Low FSSB	−0.02	0.04	[−0.09, 0.05]
	Differences	0.16	0.06	[0.07, 0.29]
W_ICTs → Challenge appraisal → Fatigue	High FSSB	−0.08	0.04	[−0.17, −0.002]
	Low FSSB	0.01	0.02	[−0.03, 0.05]
	Differences	−0.09	0.05	[−0.18, −0.002]

Note: *N* = 200. W_ICTs = work-related use of information and communication technologies, FSSB = family supportive supervisor behaviors.

## Data Availability

The data are available on reasonable request from the corresponding author.
